# Improvement in bone marrow infiltration in patients with type I Gaucher disease treated with taliglucerase alfa

**DOI:** 10.1007/s10545-018-0195-y

**Published:** 2018-07-31

**Authors:** Ari Zimran, Tama Dinur, Shoshana Revel-Vilk, Eric M. Akkerman, Laura van Dussen, Carla E. M. Hollak, Hannah Maayan, Gheona Altarescu, Raul Chertkoff, Mario Maas

**Affiliations:** 1 Gaucher Clinic, Shaare Zedek Medical Center, affiliated with Hebrew-University Medical School, Jerusalem, Israel; 2 Academic Medical Centre, Amsterdam, Netherlands; 3 Genetic Unit, Shaare Zedek Medical Center, Jerusalem, Israel; 4 Protalix Biotherapeutics, Carmiel, Israel

**Keywords:** Gaucher disease, Enzyme replacement therapy, Taliglucerase alfa, Quantitative chemical shift imaging, Bone response

## Abstract

Preliminary data suggest a positive effect of taliglucerase alfa on the bone marrow infiltration of Gaucher cells. In this investigator-initiated study, we report the impact of taliglucerase alfa on the bone marrow fat fraction (FF) in 26 patients assessed by quantitative chemical shift imaging (QCSI). Of 15 treatment-naïve patients (median age 48 [range 24–68] years), eight had baseline FF ≤ 0.3, six of those with a FF ≤ 0.23 (‘bone at risk’). All significantly improved from a median baseline FF of 0.24 (0.15–0.32) to 1st year FF of 0.37 (0.25–0.54) and 2nd year FF of 0.42 (0.27–0.59) (*p* = 0.01). Among the 11 ‘switch-over’ patients (median age 42 [range 33–69] years; median imiglucerase exposure 8 [range 1–17] years), eight had baseline FF ≤ 0.3, five of those with FF < 0.23. All, but one, significantly improved from a median baseline FF of 0.17 (0.08–0.28) to 1st year FF of 0.3 (0.05–0.34) and 2nd year FF of 0.34 (0.08–0.44) (*p* = 0.03). Two elderly female patients (age 43 and 58 years, with 17 years imiglucerase exposure) who remained at the same enzyme replacement therapy dose, increased from baseline FF of 0.13 and 0.19 to 0.26 at 1 year. Although the number of observations is small, we hypothesize that switching to taliglucerase may result in an improved bone marrow response. A larger study is needed to assess the early benefit of taliglucerase alfa in adult patients with type 1 Gaucher disease on the bone marrow compartment.

## Introduction

Gaucher disease (GD), one of the most prevalent lysosomal storage disorder, results from defective β-glucocerebrosidase production and subsequent accumulation of glucocerebroside in macrophages (Zimran and Elstein 2016). Enzyme replacement therapy (ERT) became available in 1991 (Barton et al 1991) leading to improvement in hemoglobin levels and platelet counts, and to reduction of splenic and hepatic enlargement (Weinreb et al 2002; Zimran and Elstein 2014), but the bone response lagged behind the visceral and hematological improvements (Lebel et al 2004; Wenstrup et al 2007).

Infiltration of the bone marrow by Gaucher cells reduces the bone marrow fat fraction (FF), and the reduction in lumbar spine FF is closely related to the extent of skeletal manifestations of GD (Rosenthal et al 1995; Maas et al 2002). The Dixon-quantitative chemical shift imaging (QCSI) was developed in The Academic Medical Center (AMC), Amsterdam, as a technique that measures displacement of fatty marrow by Gaucher cells using the mean value of the FF in vertebrae L3, L4, and L5 (Hollak et al 2001; van Dussen et al 2014). The FF measured in patients with GD ranged from 0.08 to 0.40 (mean 0.20), significantly lower than in the healthy population (range 0.27 to 0.55; mean 0.37; *p* < 0.001), while bone complications occurred primarily in patients with a FF of less than 0.23, indicative of “bone at risk” (Maas et al 2002).

Taliglucerase alfa is the first plant cell–expressed recombinant therapeutic protein approved for use in humans and is approved for the treatment of patients with GD in multiple countries (Zimran et al 2011; Grabowski et al 2014; Tekoah et al 2015). In 2012, we reported improvement in QCSI in a sub-group of eight patients from the pivotal taliglucerase alfa clinical trial, who were willing to travel to the AMC in Amsterdam to undergo periodic QCSI studies (van Dussen et al 2013). In this small cohort of patients treated with either 30 or 60 units/kg every other week (EOW), a significant increase in lumbar spine FF was shown, reflecting the clearance of Gaucher cells from the bone marrow. Of the eight patients, five patients had a baseline FF below 0.23. Interestingly, the improvement occurred as early as 9 months after the beginning of treatment with taliglucerase alfa, with further improvement up to 36 months (Tekoah et al 2015). Although comparable data at 9 months of therapy have not been reported for treatment with imiglucerase or velaglucerase alfa, we hypothesized that taliglucerase had a more specific beneficial effect on bone marrow involvement, and therefore we initiated a study to assess the bone marrow response to taliglucerase alfa using the same methodology of QCSI in a larger, new cohort of adult patients with GD, both treatment-naïve to ERT and those switched from imiglucerase, who received taliglucerase alfa during the 3 years of approved early access programs (EAP) in Israel.

## Methods

The study was initiated by investigators at Shaare Zedek Medical Center (SZMC), Jerusalem, Israel, and the AMC at the University of Amsterdam, Amsterdam, the Netherlands. Patients from the Gaucher Clinic at SZMC who were eligible for the EAP included those who had previously received imiglucerase (‘switch-over’) and those who had never received any ERT or who had not received ERT for ≥2 years (‘naïve’). The EAP excluded children and pregnant women (those patients continued with imiglucerase).

All patients (whether treatment-naïve to ERT or switched from imiglucerase) were offered the option to undergo QCSI at AMC before treatment initiation. Those who presented with a FF below 0.3 were offered an additional two follow-up examinations at 1 and 2 years from the advent of taliglucerase alfa therapy. Travel and QCSI costs were covered by Protalix Biotherapeutics.

Most patients in this study received taliglucerase alfa 30 units/kg EOW with the exception of four patients; three ‘naïve’ patients received 60 units/kg EOW because of significant thrombocytopenia and bleeding tendency (*n* = 1) and the need for dose increase during the study due to poor platelet response (*n* = 2) (Table 1). One ‘switch-over’ patient originally from the expanded access protocol PB-06-004 (NCT00962260) continued with taliglucerase alfa 15 U/kg EOW (Table 2). For the majority of the patients who switched from imiglucerase the 30 U/kg, EOW was, in fact, a dose increase (doubled). None of the switched patients experienced any interruption of imiglucerase ERT prior to the advent of taliglucerase alfa treatment.

**Table 1:** Demographics and lumbar spine FF assessments in treatment-naïve patients (*n* = 15)

P#	Age, yr	Gender	Taliglucerase alfa		Lumbar spine FF		
			Duration, yr	Dose, U/kg	Baseline	Year 1	Year 2
Lumbar spine FF ≤0.30							
46	66	Female	2	30	0.15	0.26	0.41
17	68	Female	3.3	30	0.16	0.25	0.27
29	48	Female	5	30	0.17	0.53	0.59
33	45	Female	4.5	30	0.21	0.36	0.35
41*	41	Female	4.5	30	0.21	–	–
32*	32	Female	0.5	30	0.23	–	–
18*	65	Female	1	30	0.27	–	–
44	47	Male	4.2	30	0.27	0.36	0.41
13	63	Male	4	60	0.30	0.39	0.44
Lumbar spine FF >0.30							
23§	46	Female	5	30	0.31	–	–
35^†^	55	Male	4.5	60^‡^	0.32	0.43	0.45
38^†^	34	Male	4	30	0.32	0.54	0.50
42^§^	24	Female	4.5	30	0.39	–	–
34^§^	65	Female	4	60^‡^	0.40	–	–
31^§^	60	Male	4.7	30	0.43	–	–

P, patient; yr., year; U, unit; FF, fat fraction

*Withdrew from the study (early withdrawal due to allergic reaction [n = 1] and personal reasons [n = 2]). ^†^Two patients with lumbar spine FF >0.30 were followed via quantitative chemical shift imaging (one due to severe osteoporosis and the other due to very severe phenotype at baseline). ^‡^Taliglucerase alfa dosing was started at 30 U/kg and increased to 60 U/kg. ^§^Not followed with QCSI because baseline lumbar spine FF was >0.30

**Table 2:** Demographics and lumbar spine FF assessments in treatment-switch patients (*n* = 11)

P #	Age, yr	Gender	Duration of imiglucerase, yr	Dose of imiglucerase, U/kg	Duration of taliglucerase alfa, yr	Dose of taliglucerase alfa, U/kg	Lumbar spine FF		
							Baseline	Year 1	Year 2
Lumbar spine FF ≤0.30									
37	42	Female	15	15	4.5	30	0.08	0.05	0.08
30	58	Female	17	30	5	30	0.13	0.29	0.44
14	35	Female	2.2	15	4	30	0.17	0.31	0.31
19	33	Female	7.6	15	4.5	30	0.17	0.15	0.26
1	43	Female	17	15	1.5	15	0.19	0.29	–
22	39	Male	14	15	4.7	30	0.24	0.34	0.36
39	44	Female	14	15	4.5	30	0.26	0.30	0.35
36	69	Male	6.5	15	4.5	30	0.28	0.34	0.34
Lumbar spine FF >0.30									
25*	34	Male	6.5	15	5	30	0.46	–	–
27*	50	Male	0.9	15	0.5	30	0.49	–	–
20*	40	Male	8	15	5	30	0.51	–	–

P, patient; yr., year; U, unit; FF, fat fraction

*Not followed by quantitative chemical shift imaging because baseline lumbar spine FF was >0.30

The QCSI was performed on a Siemens Avanto machine, as previously reported (van Dussen et al 2013). Lumbar spine FF was calculated as the average of readings for L3, L4, and L5 using a standard algorithm developed by the AMC. When obtaining consecutive measurements from patients, a special effort was taken to reposition the image slide on the mid-sagittal localizer as close as possible to obtain regions of interest for L3, L4, and L5 in a standardized manner. This protocol was reproducibile (standard deviation of repeated measurements of 0.01–0.03) (Maas et al 2001; Maas et al 2003). The QCSI technique in the AMC institute has been part of regular clinical routine for two decades and undergoes regular quality assessment (van Dussen et al 2014).

Institutional review board approval was granted for the study and all participating patients provided written informed consent before commencing study procedures.

### Statistical analysis

Descriptive statistics were employed. Absolute changes in bone marrow FF were calculated. Differences in FF compared to baseline after the 1st and 2nd year in ‘naïve’ and in ‘switch-over’ patients were tested using the related samples Wilcoxon signed ranks test. Statistical analysis was performed with SPSS statistical package (version 22 for Windows). A *P* value <0.05 was considered significant.

## Results

**Fig. 1:**
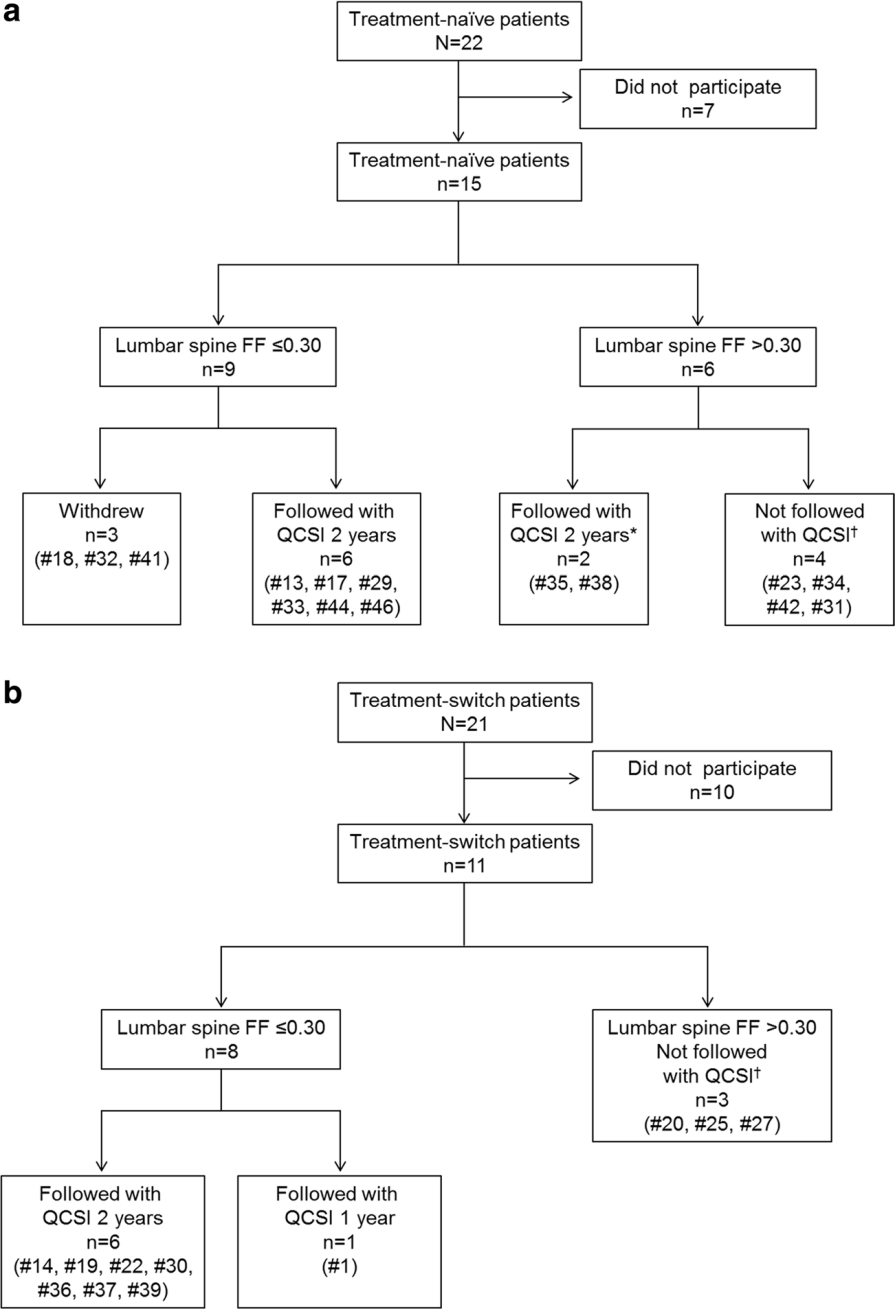
Patient disposition in the Early Access Program. *Two patients with lumbar spine fat fraction (FF) >0.30 were followed via quantitative chemical shift imaging (QCSI) (one due to severe osteoporosis and the other with very severe phenotype at baseline). ^†^Not followed with QCSI because baseline FF was >0.30

**Fig. 2:**
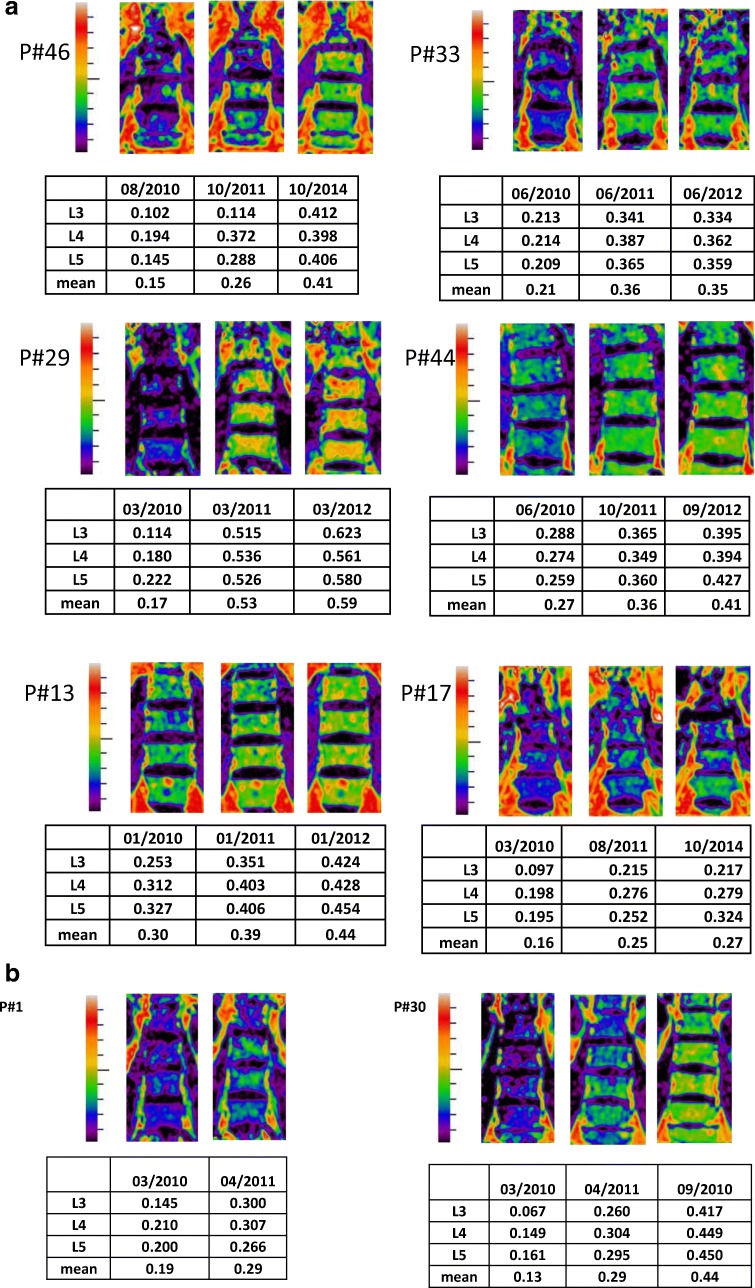
**a** Quantitative chemical shift imaging (QSCI) findings from treatment-naïve patients with lumbar spine fat fraction (FF) ≤0.30 at baseline.** b** Quantitative chemical shift imaging (QSCI) findings from treatment-switch patients who remained at the same dose

Of the 22 treatment-naïve patients who were given at least one dose of taliglucerase alfa, 15 patients agreed to participate in the study and were referred for baseline QCSI at AMC (Fig. 1). Median FF at baseline was 0.27 (range 0.16–0.43) (see Table 1). Eight patients performed follow-up QCSI assessments after the 1st and 2nd year of therapy. All patients showed significant improvement in FF as early as 1 year of taliglucerase alfa from a median baseline FF of 0.24 (0.15–0.32) to 1st year FF of 0.37 (0.25–0.54) and 2nd year FF of 0.42 (0.27–0.59) (*p* = 0.01) (Table 1). Importantly, all four treatment-naïve patients with FF < 0.23 changed FF category from “bone at risk” to “no risk”. The QCSI results from treatment-naïve patients with a baseline lumbar spine FF ≤ 0.30 are shown in Fig. 2a.

Of the 21 patients who received taliglucerase alfa after being treated with imiglucerase (‘switch-over’ patients), 11 patients agreed to participate in the study (Fig. 1b). Median FF at baseline was 0.24 (range 0.08–0.51). All eight patients with FF < 0.3 performed at least one follow up assessment (Table 2). Seven patients significantly improved over baseline from a median baseline FF of 0.17 (0.08–0.28) to 1st year FF of 0.3 (0.05–0.34) and 2nd year FF of 0.34 (0.08–0.44) (*p* = 0.035 and 0.027, respectively). One patient with FF of 0.08, the lowest in the cohort of ‘switch-over’ patients (after 15 years imiglucerase), did not increase the FF after the ‘switch-over’ despite a doubling of the dose. The improvements in QCSI parameters for the two ‘switch over’ patients who remained at the same dose (Table 2, P#30; P#1) are shown in Fig. 2b.

## Discussion

Taliglucerase alfa, the first plant-cell expressed human recombinant therapeutic protein, is the third ERT approved for the treatment of adults and children with type 1 GD (Shemesh et al 2015; Stirnemann et al 2017). Its safety and efficacy have been demonstrated in three double-blind two-dose (30 and 60 units/kg EOW) comparative clinical trials: treatment-naïve adults (Zimran et al 2016), treatment-naïve children (Zimran et al 2015), and in a switch-over trial with both adult and pediatric patients who had been previously treated with imiglucerase (Pastores et al 2014). In all these trials (as in other trials of ERTs for GD), efficacy endpoints were a reduction in spleen and liver volumes and improvement in hematological parameters, whereas the impact on bones has been considered exploratory because of the slower response of the skeleton to alglucerase and to imiglucerase (Lebel et al 2004).

During the clinical development program of taliglucerase alfa, it was speculated that the presence of 100% mannose residues compared to only 40–60% in imiglucerase (Tekoah et al 2015) could account for the better penetrance of taliglucerase alfa into bone marrow macrophages. Therefore, the option of QCSI examination was offered as an additional skeletal (exploratory) end-point.

The current report reinforces the earlier findings of clinically meaningful early improvement in bone marrow FF of the lumbar spine in patients naïve to ERT (van Dussen et al 2013). Importantly, the QSCI in ‘switch-over patients was studied here for the first time, demonstrating improvement of FF among the majority of those patients. However, because many of the switched patients had their dosage increased from 15 to 30 units/kg EOW, one cannot claim a “booster effect” in bone response by virtue of the switch alone. Several reports have already demonstrated dose-response relations in the bone changes as measured by bone densitometry (Wenstrup et al 2007) and by bone marrow burden score assessed by MRI (de Fost et al 2006). Of interest is the improvement in QCSI parameters of the two patients who remained at the same dose. These patients, age 43 and 58 years, remained at the same dose as before the switch and, despite very low FF values after 17 years on imiglucerase, changed category from “bone at risk” (0.13; 0.19, respectively) to FF = 0.29 after 1 year of taliglucerase alfa and to FF = 0.44 for one of the patients at 2 years. As conversion to fatty marrow may occur during aging, especially in peri-menopausal patients, it is not clear if this phenomenon has influenced the outcome. The female with poor response to taliglucerage alpha (#37) was most probably a poor responder with severe stable bone disease. Over the years there was no evidence for antibody formation, myelofibrosis, and/or other underlying bone disease that could explain her lack of response to the increased taliglucerase doses. We acknowledge that the small cohort and variable results should be interpreted with caution. However, if these observations would be seen in larger cohorts of patients, there may be a medical indication to switch patients with low FF values despite long-term exposure to other Gaucher-specific treatments to taliglucerase alfa.

The QCSI is considered the most sensitive and specific imaging modality to assess bone marrow involvement and response to Gaucher-specific therapy (van Dussen et al 2014); yet its very restricted availability is the main limitation of the current and future studies.

The early improvement in QCSI results observed in the treatment-naïve patients is similar to the early improvement in bone marrow burden scores evaluated in the velaglucerase alfa clinical trial (Elstein et al 2014). As both taliglucerase alfa and velaglucerase alfa share a complete mannose residues profile (100% versus 40–60% on imiglucerase), if confirmed in a larger cohort, we hypothesize that this preferred glycan structure of the enzymes may possibly be responsible for the early response of the bone marrow FF to these two newer ERTs.

The study has several limitations. In addition to the limited number of patients studied to support a booster effect of taliglucerase and the potential effect of aging on the observed improvement, we have not observed any bone events in these patients, although the general agreement is that a low FF is associated with an increased risk for complications.

In conclusion, the current report adds to the overall satisfying efficacy profile of taliglucerase alfa in adult patients with type 1 GD, and again suggests a beneficial impact on the bone marrow compartment, a parameter that has heretofore been considered slower to response compared to the hematological and visceral disease features. Further studies are needed to establish a true “booster effect” of taliglucerase on the bone marrow compartment.
